# Association between daily sunlight exposure duration and diabetic retinopathy in Korean adults with diabetes: A nationwide population-based cross-sectional study

**DOI:** 10.1371/journal.pone.0237149

**Published:** 2020-08-07

**Authors:** Hye Jun Lee, Choon Ok Kim, Duk Chul Lee

**Affiliations:** 1 Department of Family Medicine, College of Medicine, Yonsei University, Seoul, Korea; 2 Department of Clinical Pharmacology, Severance Hospital, Yonsei University Health System, Seoul, Korea; University of Utah, UNITED STATES

## Abstract

**Purpose:**

To investigate the association between daily sunlight exposure duration and diabetic retinopathy in Korean adults with diabetes.

**Methods:**

This study used data from the 2008–2011 Korea National Health and Nutrition Examination Survey. Overall, 1,089 patients with diabetes aged >40 years were included. The duration of daily sunlight exposure was assessed via health interviews. Comprehensive ophthalmic evaluations, including standard retinal fundus photography after pupil dilation, were conducted. Diabetic retinopathy was graded using the modified Airlie House Classification. Multivariate logistic regression analysis was performed to analyze the association between daily sunlight exposure duration and the diagnosis of diabetic retinopathy and non-proliferative diabetic retinopathy.

**Results:**

The risk of diabetic retinopathy was 2.66 times higher in the group with ≥5 h of daily sunlight exposure than in the group with less exposure after adjusting for risk factors such as duration of diabetes, serum hemoglobin A1c level, hypertension, and dyslipidemia (*P* = 0.023). Furthermore, the risk of non-proliferative diabetic retinopathy was 3.13 times higher in the group with ≥5 h of daily sunlight exposure than in the group with less exposure (*P* = 0.009). In patients with diabetes for <10 years, the risks of diabetic retinopathy and non-proliferative diabetic retinopathy were 4.26 and 4.82 times higher in the group with ≥5 h of daily sunlight exposure than the group with less exposure, respectively (*P* < 0.05).

**Conclusions:**

This study revealed that sunlight exposure for ≥5 h a day was significantly associated with an increased risk of diabetic retinopathy and non-proliferative diabetic retinopathy in Korean patients with diabetes. The risks were significantly higher in patients with diabetes for <10 years. Therefore, reducing daily sunlight exposure could be an early preventive strategy against diabetic retinopathy in people with diabetes.

## Introduction

Diabetes mellitus is one of the most common chronic metabolic diseases. Because of the high incidence of diabetes worldwide, the management of its chronic complications and glycemic control have become increasingly important. Diabetic retinopathy (DR) is the most common microvascular complication of diabetes and is the leading cause of blindness [1].

According to recent epidemiologic studies on DR in Korea, the prevalence of DR in patients diagnosed with diabetes is 11–19% [2]. The prevalence of severe complications such as severe macular edema and proliferative diabetic retinopathy (PDR) is approximately 5% [2]. Moreover, the risk of DR increased more than 15 times in patients with diabetes for 11 years [2–4].

The main mechanism underlying the development of DR is related to oxidative stress caused by hyperglycemia and also photooxidation of the retina owing to external environmental factors [5]. Previous studies have shown that ultraviolet (UV) radiation can exacerbate DR, especially in patients with diabetic hyperglycemia [6]. As UV radiation is present in sunlight, the amount of UV exposure increases with longer sunlight exposure [7].

In this study, we investigated the relationship between daily sunlight exposure duration and DR in Korean adults with diabetes using data from the Korea National Health and Nutrition Examination Survey (KNHANES). In addition, we analyzed differences in risk based on the duration of diabetes and types of DR. Considering that UV radiation can exacerbate DR and is dependent on the amount of sunlight exposure, we focused on quantitative figures of sunlight exposure duration that could reflect oxidative stress and photooxidation of retina as major causes of DR.

## Materials and methods

### Study population

This cross-sectional study assessed data from the 2008–2011 KNHANES. The KNHANES is a nationwide population-based survey that is being conducted by the Korean Ministry of Health and Welfare and the Division of Chronic Disease Surveillance of the Korean Centers for Disease Control and Prevention.

**Fig 1:**
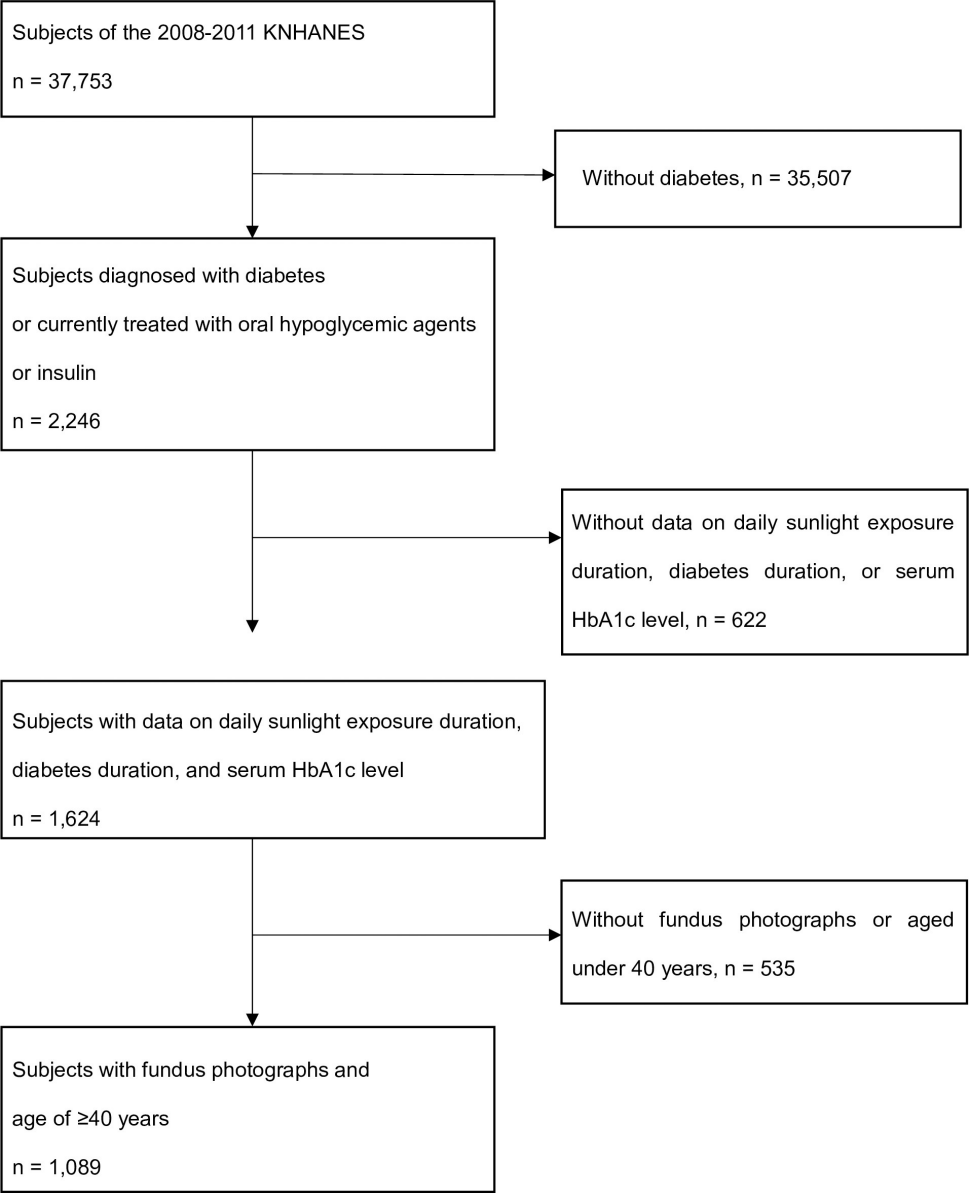
Flow diagram of the selection of study subjects. KNHANES, Korea National Health and Nutrition Examination Survey; HbA1c, hemoglobin A1c.

Among the 37,753 subjects of the 2008–2011 KNHANES, a total of 2,246 patients were diagnosed with diabetes or treated with oral hypoglycemic agents or insulin. As the incidence of diabetic complications, including DR, tends to be higher among Korean patients with diabetes who are >40 years old than among younger age groups [8], we set the age of >40 years as one of the inclusion criteria. For this study, subjects without data on daily sunlight exposure duration, duration of diabetes, or hemoglobin A1c (HbA1c) levels were excluded. Subjects aged <40 years and who had not undergone a retinal fundus examination were also excluded. Finally, a total of 1,089 subjects were included in the analysis (Fig 1).

All participants provided written informed consent before participation in the study, and the KNHANES was conducted following ethical approval by the Institutional Review Board of the Korea Centers for Disease Control and Prevention (No. 2008-04EXP-01-C, 2009-01CON-03-2C, 2010-02CON-21-C, and 2011-02CON-06C). The protocol of this study was approved by the Institutional Review Board of Yonsei University, Seoul, Korea (No. 4-2020-0121).

### Data collection

Subjects were interviewed by trained staff using standardized health questionnaires on demographic information, medical and drug history, smoking, alcohol intake, daily sunlight exposure duration, etc. Diabetes was defined as a self-report of physician-based diagnosis of diabetes or current treatment with oral hypoglycemic agents or insulin. Hypertension and dyslipidemia were also defined in the same manner with diabetes.

Seven standard photographs of both eyes were taken after pharmacological pupil dilatation according to the protocol of the Early Treatment for Diabetic Retinopathy Study in participants with a history of diabetes, a random blood glucose level of ≥200 mg/dL, or suspicious DR findings on nonmydriatic 45° digital fundus photographs (TRC-NW6S; Topcon, Tokyo, Japan). Two trained ophthalmologists blinded to the subjects’ information analyzed the fundus photographs. DR was identified if any characteristic lesion defined by the Early Treatment for Diabetic Retinopathy Study severity scale was present; these lesions included microaneurysm, hemorrhage, hard exudates, cotton wool spots, intraretinal microvascular abnormalities, venous beading, and development of new vessels (i.e., neovascularization at the disc and neovascularization elsewhere) [9]. DR was further classified into PDR and non-PDR (NPDR). A DR severity score was assigned to each eye according to a modified version of the Airlie House Classification system [10]. DR was defined as having a severity score >14. In particular, PDR was defined as a score >60 and NPDR as a score <60 [10]. As the number of PDR cases from the KNHANES data was very small (n = 17), these cases were excluded from the analysis.

Sunlight exposure duration was assessed using surveys and categorized as <5 h or ≥5 h. The duration of diabetes was calculated as the number of years since the patient was diagnosed with diabetes. Body mass index (BMI) was calculated by dividing weight (kg) by the square of height (m^2^). Blood pressure was measured three times by an experienced nurse using a sphygmomanometer with the patient in the sitting position; the average of the second and third measurements was used for analysis. Smoking was defined as current, past, or never, whereas monthly alcohol intake was defined as drinking more than once a month during the past year.

Fasting blood glucose, total cholesterol, and triglyceride levels were measured using an automatic analyzer (Hitachi 7600; Hitachi, Tokyo, Japan), and HbA1c levels were measured using an automated glycohemoglobin analyzer (HLC-723G7; Tosoh, Tokyo, Japan). Serum 25-hydroxyvitamin D levels were measured with a radioimmunoassay kit (DiaSorin Inc., Stillwater, MN, USA) using a gamma counter (1470 WIZARD; PerkinElmer Inc., Waltham, MA, USA).

### Statistical analysis

The characteristics of the subjects classified according to the presence of DR, PDR and NPDR were expressed as means and standard deviations for continuous variables and as numbers and percentages for categorical variables. They were compared using an independent t-test for continuous variables and a chi-squared test for categorical variables. Differences in daily sunlight exposure duration were evaluated using a chi-squared test based on the presence of DR, PDR and NPDR.

Multivariate logistic regression analysis was performed to analyze the association between daily sunlight exposure duration and the risk of DR and NPDR. Relative risks were estimated in terms of odds ratios (ORs) and 95% confidence intervals (CIs). We adjusted for multiple variables that showed significant associations in the univariate analysis and those based on clinical relevance. After calculating the crude ORs (Model 1), Model 2 was adjusted for age, sex, duration of diabetes, serum HbA1c level, and BMI. Model 3 was further adjusted for serum 25-hydroxyvitamin D level, hypertension, dyslipidemia, smoking, and alcohol intake. All logistic regression analysis variables were examined for multicollinearity, and only those variables with a variance inflation factor <5 were used. All statistical analyses were performed using IBM SPSS version 25 (Armonk, NY, USA). The level of statistical significance was set at *P* <0.05, and *P* values were two-tailed.

## Results

### Demographic characteristics of the subjects

A total of 1,089 subjects were included in this study (Fig 1). Table 1 shows the demographic characteristics of the subjects according to the DR classification. The prevalence rates of DR, PDR and NPDR were 21.40% (n = 233), 1.56% (n = 17), and 19.83% (n = 216), respectively. The mean age was 63.49 ± 9.33 years, and the number of males was 546 (50.1%).

**Table 1:** Demographic characteristics according to the presence of diabetic retinopathy and proliferative diabetic retinopathy in Korean adults with diabetes.

Characteristic	DR		*P* value	PDR		*P* Value	NPDR		*P* Value	Total (n = 1089)
	No (n = 856)	Yes (n = 233)		No (n = 1072)	Yes (n = 17)		No (n = 873)	Yes (n = 216)		
**Age (years)**	63.45 ± 9.37	63.65 ± 9.18	0.775	63.53 ± 3.53	61.53 ± 1.53	0.379	63.42 ± 9.36	63.81 ± 9.19	0.570	63.49 ± 9.33
**Male (%)**	431 (50.4)	115 (49.4)	0.788	537 (50.1)	9 (52.9)	0.816	440 (50.4)	106 (49.1)	0.727	546 (50.1)
**BMI (kg/m**^**2**^**)**	24.91 ± 3.34	23.82 ± 2.86	<0.001	24.70 ± 4.70	22.89 ± 2.89	0.034	24.87 ± 3.35	23.89 ± 2.82	<0.001	24.67 ± 3.27
**HbA1c (%)**	7.20 ± 1.33	8.15 ± 1.62	<0.001	7.39 ± 3.91	8.26 ± 2.61	0.014	7.22 ± 1.35	8.14 ± 1.60	<0.001	7.40 ± 1.45
**FBG (mg/dL)**	142.85 ± 55.82	165.41 ± 55.84	<0.001	150.33 ± 56.86	165.00 ± 54.86	0.380	137.43 ± 47.51	159.57 ± 53.20	<0.001	141.82 ± 49.46
**Vit D (ng/mL)**	19.36 ± 9.36	18.81 ± 6.80	0.279	19.28 ± 9.28	16.94 ± 66.53	0.161	19.32 ± 7.11	18.96 ± 6.81	0.497	19.25 ± 7.05
**TChol (mg/dL)**	180.74 ± 37.82	189.08 ± 44.56	0.004	182.51 ± 82.51	182.94 ± 82.94	0.968	180.78 ± 37.92	189.56 ± 44.66	0.003	182.52 ± 39.48
**TG (mg/dL)**	163.94 ± 106.57	188.11 ± 88.11	0.017	169.48 ± 69.48	145.06 ± 45.06	0.158	163.58 ± 105.94	191.51 ± 221.65	0.007	169.10 ± 137.11
**SBP (mmHg)**	126.44 ± 16.82	128.72 ± 28.72	0.075	126.88 ± 26.88	130.39 ± 30.39	0.515	126.52 ± 16.92	128.59 ± 18.89	0.142	126.93 ± 17.34
**DBP (mmHg)**	74.96 ± 9.87	73.08 ± 10.45	0.015	74.58 ± 10.05	72.98 ± 8.23	0.439	74.92 ± 9.83	73.09 ± 10.62	0.022	74.56 ± 10.02
**DM duration (years)**	7.47 ± 7.30	12.14 ± 8.77	<0.001	8.36 ± 3.61	15.24 ± 5.24	0.003	7.62 ± 7.46	11.89 ± 8.53	<0.001	8.47 ± 7.87
** <10 years**	601 (70.2)	95 (40.8)		691 (64.5)	5 (29.4)		606 (69.4)	90 (41.7)		696 (63.9)
** ≥10 years**	255 (29.8)	138 (59.2)		381 (35.5)	12 (70.6)		267 (30.6)	126 (58.3)		393 (36.1)
**Hypertension**^†^	524 (99.4)	119 (97.5)	0.049	634 (99.1)	9 (100.0)	0.770	533 (99.4)	110 (97.3)	0.034	643 (99.1)
**Dyslipidemia**^‡^	268 (97.8)	65 (98.5)	0.729	328 (97.9)	5 (100.0)	0.774	273 (97.8)	60 (98.4)	0.799	333 (97.9)
**Smoking**			0.195			0.331			0.175	
** Current**	164 (19.2)	54 (23.2)		216 (20.2)	2 (11.8)		166 (19.0)	52 (24.1)		218 (20.0)
** Past**	242 (28.3)	54 (23.2)		293 (27.4)	3 (17.6)		245 (28.1)	51 (23.6)		296 (27.2)
** Never**	449 (52.5)	125 (53.6)		562 (52.5)	12 (70.6)		461 (52.9)	113 (52.3)		574 (52.8)
**Monthly alcohol intake**			0.216			0.286			0.348	
** Yes**	366 (43.1)	89 (38.5)		450 (42.3)	5 (29.4)		371 (42.8)	84 (39.3)		455 (42.1)
** No**	484 (56.9)	142 (61.5)		614 (57.7)	12 (70.6)		496 (57.2)	130 (60.7)		626 (57.9)
**Daily sunlight exposure**			0.741			0.698			0.826	
** <5 h**	641 (74.9)	172 (73.8)		801 (74.7)	12 (70.6)		653 (74.8)	160 (74.1)		813 (74.7)
** ≥5 h**	215 (25.1)	61 (26.2)		271 (25.3)	5 (29.4)		220 (25.2)	56 (25.9)		276 (25.3)

DR, diabetic retinopathy; PDR, proliferative diabetic retinopathy; NPDR, non-proliferative diabetic retinopathy; BMI, body mass index; HbA1c, hemoglobin A1c; FBG; fasting blood glucose; Vit D, 25-hydroxyvitamin D; TChol, total cholesterol; TG, triglycerides; SBP; systolic blood pressure; DBP, diastolic blood pressure; DM, diabetes mellitus.

Data from the 2008–2011 Korean National Health and Nutrition Examination Survey.

*P* values were calculated using an independent t-test or chi-squared test.

Continuous variables are expressed as means and standard deviations, and categorical variables are expressed as numbers and percentages.

^**†**^Hypertension was defined as a self-report of physician-based diagnosis of hypertension or current treatment with antihypertensive medication.

^**‡**^Dyslipidemia was defined as a self-report of physician-based diagnosis of dyslipidemia or current treatment with antidyslipidemic medication.

Serum HbA1c, fasting blood glucose, total cholesterol, and triglyceride levels were higher in the DR and NPDR groups than in the group without any DR or NPDR, whereas BMI and diastolic blood pressure were lower in the DR and NPDR groups (all *P* < 0.05). In addition, the duration of diabetes was longer and hypertension was more prevalent in subjects with DR and NPDR (all *P* < 0.05). However, there were no significant differences in age, serum 25-hydroxyvitamin D levels, systolic blood pressure, dyslipidemia, daily sunlight exposure duration, smoking, and alcohol intake between the two groups (all *P* > 0.05).

Table 2 shows the demographics associated with each variable according to daily sunlight exposure duration. The group exposed to sunlight for ≥5 h a day had 167 males (60.5%) and had higher serum 25-hydroxyvitamin D levels, lower total cholesterol levels, and higher prevalence rates of smoking and alcohol intake than the group exposed to sunlight for <5 h (all *P* < 0.05).

**Table 2:** Demographic characteristics according to daily sunlight exposure duration in Korean adults with diabetes.

Variable	Daily Sunlight Exposure Duration		*P* Value
	<5 h (n = 813)	≥5 h (n = 276)	
**Age (years)**	63.65 ± 9.44	63.03 ± 8.97	0.329
**Male (%)**	379 (46.6)	167 (60.5)	<0.001
**BMI (kg/m**^**2**^**)**	24.75 ± 3.28	24.45 ± 3.24	0.184
**HbA1c (%)**	7.40 ± 1.46	7.39 ± 1.41	0.924
**FBG (mg/dL)**	140.65 ± 48.47	145.26 ± 52.19	0.181
**Vit D (ng/mL)**	18.70 ± 7.01	20.87 ± 6.92	<0.001
**TChol (mg/dL)**	184.15 ± 40.15	177.67 ± 37.07	0.018
**TG (mg/dL)**	171.38 ± 145.69	162.35 ± 107.79	0.345
**SBP (mmHg)**	127.10 ± 17.09	126.43 ± 18.06	0.588
**DBP (mmHg)**	74.65 ± 9.91	74.27 ± 10.36	0.594
**DM duration (years)**	8.47 ± 7.90	8.46 ± 7.78	0.980
** <10 years**	516 (63.5)	180 (65.2)	
** ≥10 years**	297 (36.5)	96 (34.8)	
**Hypertension**^†^	492 (99.2)	151 (98.7)	0.572
**Dyslipidemia**^‡^	273 (98.2)	60 (96.8)	0.474
**Smoking**			0.005
** Current**	145 (17.9)	73 (26.4)	
** Past**	220 (27.1)	76 (27.5)	
** Never**	447 (55.0)	127 (46.0)	
**Monthly alcohol intake**			0.008
** Yes**	320 (39.8)	135 (48.9)	
** No**	485 (60.2)	141 (51.1)	
**DR**	172 (21.2)	61 (22.1)	0.741
**PDR**	12 (1.5)	5 (1.8)	0.698
**NPDR**	160 (19.7)	56 (20.3)	0.826

BMI, body mass index; HbA1c, hemoglobin A1c; FBG; fasting blood glucose; Vit D, 25-hydroxyvitamin D; TChol, total cholesterol; TG, triglycerides; SBP; systolic blood pressure; DBP, diastolic blood pressure; DM, diabetes mellitus; DR, diabetic retinopathy; PDR, proliferative diabetic retinopathy; NPDR, non-proliferative diabetic retinopathy.

Data from the 2008–2011 Korean National Health and Nutrition Examination Survey.

*P* values were calculated using an independent t-test or chi-squared test.

Continuous variables are expressed as means and standard deviations, and categorical variables are expressed as numbers and percentages.

^**†**^Hypertension was defined as a self-report of physician-based diagnosis of hypertension or current treatment with antihypertensive medication.

^**‡**^Dyslipidemia was defined as a self-report of physician-based diagnosis of dyslipidemia or current treatment with antidyslipidemic medication.

### Association between daily sunlight exposure duration and DR

Table 3 shows the ORs and 95% CIs for DR and NPDR according to daily sunlight exposure duration in 1,089 subjects with diabetes aged >40 years. The model was adjusted for age, sex, duration of diabetes, serum HbA1c level, serum 25-hydroxyvitamin D level, BMI, hypertension, dyslipidemia, smoking, and alcohol intake. As a result, the risk of DR was 2.66 times higher in the group with ≥5 h of daily sunlight exposure than in the group with <5 h of exposure (95% CI 1.14–6.21 for Model 3). Moreover, the risk of NPDR was 3.13 times higher in the group with ≥5 h of daily sunlight exposure than in the group with <5 h of exposure (95% CI 1.33–7.40 for Model 3).

**Table 3:** Unadjusted and adjusted odds ratios and 95% confidence intervals for diabetic retinopathy and non-proliferative diabetic retinopathy according to daily sunlight exposure duration in Korean adults with diabetes.

Multivariate Model	Model 1 OR (95% CI)	Model 2 OR (95% CI)	Model 3 OR (95% CI)
**DR**			
** **Sunlight exposure <5 h	Reference	Reference	Reference
** **Sunlight exposure ≥5 h	2.33 (1.09–4.97) *P* = 0.029	2.63 (1.14–6.09) *P* = 0.024	2.66 (1.14–6.21) *P* = 0.023
**NPDR**			
** **Sunlight exposure <5 h	Reference	Reference	Reference
** **Sunlight exposure ≥5 h	2.74 (1.27–5.92) *P* = 0.010	3.13 (1.34–7.32) *P* = 0.008	3.13 (1.33–7.40) *P* = 0.009

OR, odds ratio; CI, confidence interval; DR, diabetic retinopathy; NPDR, non-proliferative diabetic retinopathy.

Data from the 2008–2011 Korean National Health and Nutrition Examination Survey.

Model 1 was crude.

Model 2 was adjusted for age, sex, duration of diabetes, hemoglobin A1c level, and body mass index.

Model 3 was adjusted for serum 25-hydroxyvitamin D level, hypertension, dyslipidemia, smoking, and alcohol intake in addition to the variables adjusted in Model 2.

### Association between daily sunlight exposure duration and DR based on a 10-year duration of diabetes

Table 4 shows the ORs and 95% CIs for DR and NPDR according to daily sunlight exposure duration divided by a 10-year duration of diabetes. Among the 1,089 subjects, 393 (36.1%) were diagnosed with diabetes for >10 years. This model was also adjusted for age, sex, duration of diabetes, serum HbA1c level, serum 25-hydroxyvitamin D level, BMI, hypertension, dyslipidemia, smoking, and alcohol intake.

**Table 4:** Unadjusted and adjusted odds ratios and 95% confidence intervals for diabetic retinopathy and non-proliferative diabetic retinopathy according to the daily sunlight exposure duration divided by the duration of diabetes in Korean adults.

Multivariate Model	Model 1 OR (95% CI)	Model 2 OR (95% CI)	Model 3 OR (95% CI)
**Diabetic duration <10 years**			
** DR**			
** **Sunlight exposure <5 h	Reference	Reference	Reference
** **Sunlight exposure ≥5 h	2.18 (0.75–6.33) *P* = 0.150	3.58 (1.08–11.88) *P* = 0.037	4.26 (1.20–15.16) *P* = 0.025
** NPDR**			
** **Sunlight exposure <5 h	Reference	Reference	Reference
** **Sunlight exposure ≥5 h	2.37 (0.81–6.95) *P* = 0.115	3.86 (1.14–13.02) *P* = 0.030	4.82 (1.32–17.59) *P* = 0.017
**Diabetic duration ≥10 years**			
** DR**			
** **Sunlight exposure <5 h	Reference	Reference	Reference
** **Sunlight exposure ≥5 h	1.26 (0.64–2.50) *P* = 0.507	1.09 (0.53–2.25) *P* = 0.825	1.20 (0.56–2.60) *P* = 0.636
** NPDR**			
** **Sunlight exposure <5 h	Reference	Reference	Reference
** **Sunlight exposure ≥5 h	1.32 (0.66–2.65) *P* = 0.440	1.16 (0.56–2.40) *P* = 0.691	1.21 (0.55–2.62) *P* = 0.638

OR, odds ratio; CI, confidence interval; DR, diabetic retinopathy; NPDR, non-proliferative diabetic retinopathy.

Data from the 2008–2011 Korean National Health and Nutrition Examination Survey.

Model 1 was crude.

Model 2 was adjusted for age, sex, duration of diabetes, hemoglobin A1c level, and body mass index.

Model 3 was adjusted for serum 25-hydroxyvitamin D level, hypertension, dyslipidemia, smoking, and alcohol intake in addition to the variables adjusted in Model 2.

In patients with diabetes for >10 years, daily sunlight exposure was not significantly associated with the risk for DR or NPDR. However, in patients with diabetes for <10 years, the risk of DR was 4.26 times higher in the group with ≥5 h of daily sunlight exposure than in the group with less exposure (95% CI 1.20–15.16 for Model 3). In the case of NPDR, when the duration of diabetes was <10 years, the risk of NPDR was 4.82 times higher in the group with ≥5 h of daily sunlight exposure than in the group with less exposure (95% CI 1.32–17.59 for Model 3).

## Discussion

This cross-sectional study of data from the 2008–2011 KNHANES showed that a longer sunlight exposure of ≥5 h increased the risk of DR and NPDR in Korean patients with diabetes. In particular, a duration of diabetes of <10 years was associated with higher risks of DR and NPDR in the group that was exposed to sunlight for ≥5 h than in the group that was exposed to sunlight for <5 h.

To our knowledge, this is the first study to confirm a direct association between sunlight exposure duration and DR. In this study, serum 25-hydroxyvitamin D levels were significantly higher in patients with daily sunlight exposure of ≥5 h than in those with less exposure. The risks of DR and NPDR also increased in patients exposed to sunlight for ≥5 h. These findings suggest that in addition to the effects of serum 25-hydroxyvitamin D on DR, sunlight exposure may affect DR in other ways.

Oxidative stress is thought to be a major cause of the pathogenesis of DR [5]. As the retina is the most metabolically activated tissue in the body, it is easily affected by diabetic conditions and is vulnerable to oxidative stress [11]. Exogenous sources of oxidative stress include environmental toxins such as UV radiation from sunlight and smoke [5]. Especially during sunlight exposure, UV A light (wavelength 320–400 nm) is absorbed by the eyes, passing through all optic media to produce reactive oxygen species and cause mitochondrial dysfunction, DNA damage, and apoptotic activity of retinal pigment epithelial cells. These conditions can eventually lead to irreversible cellular necrosis of the retina [12–16]. The accumulation of reactive oxygen species can also damage blood vessels surrounding the retina, causing retinal capillary apoptosis, hypoxia, and neovascularization [17–20]. Therefore, reducing sunlight exposure by wearing protective equipment such as sunglasses can be a useful, inexpensive, and convenient way to prevent DR by reducing phototoxicity (photooxidation damage) and oxidative stress on the retina. According to a previous study, wearing sunglasses with yellow UV light protective lenses can block short wavelengths of light in patients with diabetes, reduce scatter, and increase contrast, resulting in an improved subjective vision [21]. Furthermore, a study on cataracts showed that proper UV-filtering glasses can prevent the progression of photo-induced lens damage in patients with pre-cataractous molecular changes in the lens [22, 23].

Our study also suggests the importance of early prevention of DR in patients with diabetes. We found that the risk of DR from exposure to UV radiation was higher in patients diagnosed with diabetes for <10 years. The exact mechanism is unknown. However, previous studies [24–26] showed a high incidence of microvascular complications, including DR, in patients who had diabetes for <10 years. The increased incidence was due to lower medication compliance, poor glycemic control, and a lower rate of complication screening, which are consistent with our findings. Therefore, DR prevention is very important in patients with diabetes.

This study had some limitations. First, this was a cross-sectional study, so it could not assess the causal relationship between daily sunlight exposure duration and DR. A prospective longitudinal study with a large sample must be conducted to verify this relationship. Second, a self-reported questionnaire was used to assess the duration of sun exposure during the day, which served as our basis for measuring UV exposure. There may be differences in the absorption of sunlight among different races and factors such as frequency of UV exposure, the use of sun protection, occupation, season, and latitude [27]. Therefore, further studies with quantitative methods for measuring the degree of UV exposure are required.

Nevertheless, this is the first study to quantitatively investigate the association between sunlight exposure duration and DR in patients with diabetes. By presenting environmental factors, we suggest that sunlight exposure could be a novel risk factor for DR and provide an important theoretical basis for various treatments and preventive approaches for DR. The importance of early prevention, by reducing sunlight exposure, is also suggested because of the increased risk of DR even in patients with a relatively short duration of diabetes, based on our findings. The implications of our findings could be further developed in future prospective and longitudinal studies.

In conclusion, this quantitative cross-sectional analysis of data from the 2008–2011 KNHANES revealed that longer sunlight exposure in patients with diabetes aged >40 years was associated with higher risks of DR and NPDR. The risks of DR and NPDR were particularly higher by 2.66 and 3.13 times, respectively, in the group with daily sunlight exposure of ≥5 h than in the group with less sunlight exposure (*P* = 0.023 and 0.009). Additionally, even if the duration of diabetes was <10 years, the risks of DR and NPDR were higher by 4.26 and 4.82 times, respectively, in patients exposed to sunlight for ≥5 h daily than in those exposed to sunlight for <5 h daily (*P* < 0.05). Hence, reducing the daily levels of sunlight exposure could be an early preventive strategy against DR in individuals with diabetes.

## References

[1] Cusi K, Ocampo GL. Unmet needs in Hispanic/Latino patient with type 2 diabetes mellitus. American Journal of Medicine. 2011;124(Suppl 10):S2–S9. 10.1016/j.amjmed.2011.07.017 21939795

[2] Yang JY, Kim NK, Lee YJ. Prevalence and factors associated with diabetic retinopathy in a Korean adult population: the 2008–2009 Korea National Health and Nutrition Examination Survey. Diabetes Res Clin Pract. 2013;102:218–224. 10.1016/j.diabres.2013.10.016 24268633

[3] Park CY, Park SE, Bae JC. Prevalence of and risk factors for diabetic retinopathy in Koreans with type II diabetes: baseline characteristics of Seoul Metropolitan City-Diabetes Prevention Program (SMC-DPP) participants. Br J Ophthalmol. 2012;96:151–155. 10.1136/bjo.2010.198275 21775765

[4] Kim YJ, Kim JG, Lee JY, et al Development and progression of diabetic retinopathy and associated risk factors in Korean patients with type 2 diabetes: the experience of a tertiary center. J Korean Med Sci. 2014;29:1699–1705. 10.3346/jkms.2014.29.12.1699 25469073 PMC4248594

[5] Oduntan OA, Mashige KP. A review of the role of oxidative stress in the pathogenesis of eye diseases. S Afr Optom. 2011;70:191–199.

[6] Melo T, Silva EM, Simões C, Domingues P, Domingues MR. Photooxidation of glycated and non-glycated phosphatidylethanolamines monitored by mass spectrometry. J Mass Spectrom. 2013;48(1);68–78. 10.1002/jms.3129 23303749

[7] Grandahl K, Mortensen OS, Sherman DZ, et al Solar UV exposure among outdoor workers in Denmark measured with personal UV-B dosimeters: technical and practical feasibility. Biomed Eng Online. 2017;16(1);119 10.1186/s12938-017-0410-3 29017484 PMC5634959

[8] Lee TH, Ryu HJ, Chung PW, Lim WS, Chung MY. The prevalence of diabetic complications in Korea. Korean J Intern Med. 1987; 2(1): 42–47. 10.3904/kjim.1987.2.1.42 3154816 PMC4534914

[9] Wong TY, Klein R, Islam FA, Cotch MF, Folsom AR, et al Diabetic retinopathy in a multi-ethnic cohort in the United States. American journal of ophthalmology 2006:141;446 10.1016/j.ajo.2005.08.063 16490489 PMC2246042

[10] Group diabetic retinopathy study. A modification of the Airlie House classification of diabetic retinopathy. Investigative ophthalmology & visual science 1981:21;6. 7195893

[11] Kagan V. E., Shvedova A. A., Novikov K. N., Kozlov Y. P. Light-induced free radical oxidation of membrane lipids in photoreceptors off rogretina. Biochimicaet Biophysica Acta Biomembranes. 1973;330(1):76–79. 10.1016/0005-2736(73)90285-x 4543474

[12] Atlasz T., Szabadfi K., Kiss P., Marton Z., Griecs M., Hamza L. Effects of PACAP in UVA radiation. Induced retinal degeneration models in rats. J. Mol. Neurosci. 2011;43:51 10.1007/s12031-010-9392-3 20521124

[13] Roduit R, Schorderet DF. MAP kinase pathways in UV-induced apoptosis of retinal pigment epithelium ARPE19 cells. Apoptosis. 2008;13:343–353. 10.1007/s10495-008-0179-8 18253836

[14] Hanus J, Zhang H, Wang Z, Liu Q, Zhou Q, Wang S. Induction of necrotic cell death by oxidative stress in retinal pigment epithelial cells. Cell Death Dis. 2013;4:e965 10.1038/cddis.2013.478 24336085 PMC3877549

[15] Tringali G, Sampaolese B, Clementi ME. Expression of early and late cellular damage markers by ARPE-19 cells following prolonged treatment with UV-A radiation. Mol Med Rep. 2016;14:3485–3489. 10.3892/mmr.2016.5649 27573029

[16] Miyamoto S., Martinez G. R., Medeiros M. H., Di Mascio P. Singlet molecular oxygen generated from lipid hydroperoxides by the Russell mechanism: studies using 18(O)-labeled linoleic acid hydroperoxide and monomol light emission measurements. J. Am. Chem. Soc. 2003;125:61–72. 10.1021/ja029115o 12785849

[17] Kowluru R.A., Koppolu P. Diabetes-induced activation of caspase-3 in retina: effect of antioxidant therapy. Free Radical Research. 2002;36(9):993–999. 10.1080/1071576021000006572 12448825

[18] Kowluru R. A., Koppolu P., Chakrabarti S., Chen S. Diabetes-induced activation of nuclear transcriptional factor in the retina, and its inhibition by antioxidants. Free Radical Research. 2003;37(11):1169–1180. 10.1080/10715760310001604189 14703729

[19] Du Y., Veenstra A., Palczewski K., Kern T.S. Photoreceptor cells are major contributors to diabetes-induced oxidative stress and local inflammation in the retina. Proceedings of the National Academy of Sciences of the United States of America. 2013;110(41):16586–16591. 10.1073/pnas.1314575110 24067647 PMC3799310

[20] Berkowitz B. A., Grady E. M., Khetarpal N., Patel A., Roberts R. Oxidative stress and light-evoked responses of the posterior segment in a mouse model of diabetic retinopathy. Investigative Ophthalmology & Visual Science. 2015;56(1):606–615. 25574049 10.1167/iovs.14-15687 PMC4309313

[21] Maser RE, Lenhard MJ, Frattarola J, DeCherney GS. Over-the-counter yellow ultraviolet light protective lenses: any benefit for individuals with diabetes mellitus?. Del Med J. 1999;71(7):287–290. 10457664

[22] Tidake P. Rohit Agrawal. Effect of UV Light on Diabetic Retinopathy in Pseudophakic Eye. IOSR Journal of Dental and Medical Sciences. 2018; 17(7):25–28.

[23] Lerman S, Mandal K, Misra B, Schechter A, Schenck J. Phototoxicity involving the ocular lens: in vivo and in vitro studies. Photochem Photobiol. 1991;53(2):243–247. 10.1111/j.1751-1097.1991.tb03929.x 2011629

[24] Song SH, Hardisty CA. Early onset type 2 diabetes mellitus: a harbinger for complications in later years–clinical observation from a secondary care cohort. QJM 2009;102:799–806. 10.1093/qjmed/hcp121 19734298

[25] Amutha A, Anjana RM, Venkatesan U, et al Incidence of complications in young-onset diabetes: Comparing type 2 with type 1 (the young diab study). Diabetes Res Clin Pract. 2017;123:1–8. 10.1016/j.diabres.2016.11.006 27912129

[26] Hillier TA, Pedula KL. Complications in young adults with early-onset type 2 diabetes: losing the relative protection of youth. Diabetes Care 2003;26:2999–3005. 10.2337/diacare.26.11.2999 14578230

[27] Busch EM, Gorgels TG, van Norren D. Temporal sequence of changes in rat retina after UV-A and blue light exposure. Vision Res. 1999; 39(7):1233–1247. 10.1016/s0042-6989(98)00233-8 10343838

